# The Role of Prognostic and Predictive Biomarkers for Assessing Cardiovascular Risk in Chronic Kidney Disease Patients

**DOI:** 10.1155/2020/2314128

**Published:** 2020-10-08

**Authors:** Michele Provenzano, Michele Andreucci, Luca De Nicola, Carlo Garofalo, Yuri Battaglia, Silvio Borrelli, Ida Gagliardi, Teresa Faga, Ashour Michael, Pasquale Mastroroberto, Giuseppe Filiberto Serraino, Noemi Licastro, Nicola Ielapi, Raffaele Serra

**Affiliations:** ^1^Renal Unit, “Magna Graecia”, Department of Health Sciences, “Magna Graecia”, University of Catanzaro, Italy, Catanzaro, Italy; ^2^Renal Unit, University of Campania “Luigi Vanvitelli”, Naples, Italy; ^3^Nephrology and Dialysis Unit, St. Anna University Hospital, Ferrara, Italy; ^4^Department of Experimental and Clinical Medicine “Magna Graecia”, University of Catanzaro, Italy, Catanzaro, Italy; ^5^School of Medicine, University Federico II Naples, Naples, Italy; ^6^“Sapienza” University of Rome, Department of Public Health and Infectious Disease, Roma, Italy; ^7^Interuniversity Center of Phlebolymphology (CIFL), “Magna Graecia” University of Catanzaro, Catanzaro, Italy; ^8^Department of Medical and Surgical Sciences “Magna Graecia” University of Catanzaro, Catanzaro, Italy

## Abstract

Chronic kidney disease (CKD) is currently defined as the presence of proteinuria and/or an eGFR < 60 mL/min/1.73m^2^ on the basis of the renal diagnosis. The global dimension of CKD is relevant, since its prevalence and incidence have doubled in the past three decades worldwide. A major complication that occurs in CKD patients is the development of cardiovascular (CV) disease, being the incidence rate of fatal/nonfatal CV events similar to the rate of ESKD in CKD. Moreover, CKD is a multifactorial disease where multiple mechanisms contribute to the individual prognosis. The correct development of novel biomarkers of CV risk may help clinicians to ameliorate the management of CKD patients. Biomarkers of CV risk in CKD patients are classifiable as prognostic, which help to improve CV risk prediction regardless of treatment, and predictive, which allow the selection of individuals who are likely to respond to a specific treatment. Several prognostic (cystatin C, cardiac troponins, markers of inflammation, and fibrosis) and predictive (genes, metalloproteinases, and complex classifiers) biomarkers have been developed. Despite previous biomarkers providing information on the pathophysiological mechanisms of CV risk in CKD beyond proteinuria and eGFR, only a minority have been adopted in clinical use. This mainly depends on heterogeneous results and lack of validation of biomarkers. The purpose of this review is to present an update on the already assessed biomarkers of CV risk in CKD and examine the strategies for a correct development of biomarkers in clinical practice. Development of both predictive and prognostic biomarkers is an important task for nephrologists. Predictive biomarkers are useful for designing novel clinical trials (enrichment design) and for better understanding of the variability in response to the current available treatments for CV risk. Prognostic biomarkers could help to improve risk stratification and anticipate diagnosis of CV disease, such as heart failure and coronary heart disease.

## 1. Introduction

According to the latest classification, edited by the Kidney Disease: Improving Global Outcomes Work Group (KDIGO) in 2012, chronic kidney disease (CKD) is defined as the presence of a reduced kidney function (i.e., an estimated glomerular filtration rate (eGFR) < 60 mL/min/1.73m^2^) and/or albuminuria, a strong marker of kidney damage [1]. The cause of CKD was also included in the KDIGO classification, since different causes are associated with disparate outcomes and need specific treatments [1, 2]. An important aspect that has drawn attention to this topic, in the past decades, is the global impact of CKD. The 2017 Global Burden of Disease study has shown that the number of deaths attributable to CKD increased by 33.7% over the 2007-2017 period and that this trend was higher than that of mortality due to neoplasms (+25.4%) and cardiovascular diseases (+21.1%) and close to that of diabetes mellitus (+34.7%) [3]. These general epidemiologic evidences are even more impressive when considering that from 1990 to 2016 the incidence and prevalence substantially doubled worldwide, rising by 88.76% and 86.95%, respectively [4]. The main reasons that have been considered to explain the increase in CKD burden are population growth and aging together with the decrease in age-standardized mortality and morbidity rates in most regions. Furthermore, the tide of type 2 diabetes in high-income countries has also driven the increasing trend of CKD and was confirmed as a leading cause of CKD and the more severe clinical condition of end-stage kidney disease (ESKD) [4, 5]. The result of the global dimension, when translated into clinical practice, is that a growing number of patients are exposed to both severe cardiovascular (CV) and renal risks [6, 7]. In the attempt to improve the management of CKD patients as well as to optimize the individual treatment, a large number of studies have been carried out in the past decades. Indeed, observational analyses have provided clinicians with important evidence on the predictors of poor prognosis in CKD patients, thus improving their risk stratification [8–10]. In addition, a large number of intervention studies testing the effect of antihypertensive drugs, diuretics, albuminuria-lowering agents, sodium-glucose cotransporter 2 inhibitors (SGLT-2i), and endothelin receptor antagonists on CV risk reduction in CKD patients have been conducted [11–14]. However, despite the relevant protective effects that these drugs exert against CV events, they also showed a large variability in individual response, thus determining that a considerable proportion of patients do not respond to the scheduled treatment and remain at very high risk of developing CV events [15]. To overcome individual response variability and to reduce the residual CV risk in CKD patients, several strategies have been adopted in clinical research. The first consists in designing new clinical trials that allow to understand what patient is likely to respond to a specific treatment (ClinicalTrials.gov identifier: NCT03504566), whereas the second is focusing attention on the identification, validation, and implementation of novel CV risk biomarkers that may improve risk stratification of CKD patients and identify aspects of renal disease that are not detected by albuminuria or eGFR such as inflammation, tubular damage, and fibrosis. In general, the term biomarker refers to a defined characteristic that can be measured accurately and reproducibly and evaluated as an indicator of normal biological processes, pathogenic processes, or responses to an exposure or intervention, including therapeutic interventions [16]. They have been investigated and also used for several diseases or pathologic conditions, including the renal ones [17–19]. The aim of this review is to summarize the strong association between CKD and CV disease and to examine the role of novel biomarkers of CV risk in CKD, dealing with biomarkers' function, clinical application, and future perspectives.

## 2. Cardiovascular Disease in CKD Patients

**Figure 1:**
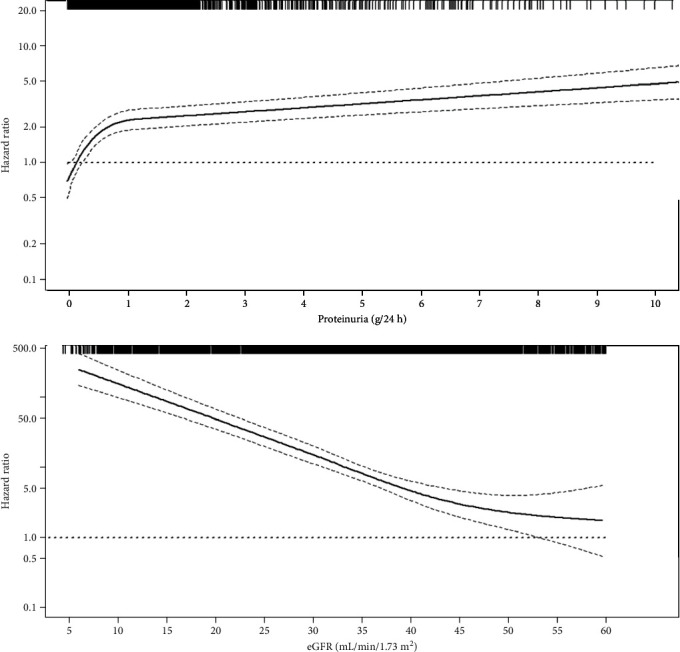
Adjusted associations between eGFR, proteinuria, and risk for cardiovascular (CV) fatal and nonfatal events (i.e., myocardial infarction, congestive heart failure, stroke, revascularization, peripheral vascular disease, nontraumatic amputation, or CV death). Solid line represents hazard ratio (HR), whereas dashed lines represent the 95% confidence intervals. HR is adjusted for the main predictors of CV events (age, gender, type 2 diabetes, history of cardiovascular disease, body mass index, hemoglobin, smoking habit, systolic blood pressure, serum phosphorus, and use of RAAS inhibitors). Knots were located at the 25^th^, 50^th^, and 75^th^ percentiles for both proteinuria and eGFR. Rug plots at the top of the *x*-axis represent the distribution of observations. Data source: CKD Multicohort, a pooled analysis of 3,957 patients referred to Italian nephrology clinics [8].

The association between CV abnormalities and CKD is an old concept. Indeed, the first scientist who described the interconnection between heart failure and the degree of renal fibrosis was Richard Bright in 1836, in a fascinating manuscript that is still available in PubMed [20]. Many studies have since confirmed this association, and explanations have been sought in terms of epidemiology, pathophysiology, and clinical perspective. From observational analyses emerged that either low eGFR or increased proteinuria, which are considered the two main kidney measures, is associated with the onset of CV complications, such as CV mortality, heart failure (HF), coronary heart disease (CAD), and stroke (Figure 1) [5, 10, 21–23]. Although results of previous studies are controversial, a recent individual-level meta-analysis of the CKD Prognosis Consortium provided strong evidence by analyzing uniformly more than 600,000 CKD patients [21]. In that meta-analysis, both eGFR and proteinuria (measured as albumin-to-creatinine ratio) predicted CV endpoints even after accounting for traditional risk factors (i.e., blood pressure, serum cholesterol levels, smoking habit, age, and gender). Interestingly, the contribution of either eGFR or proteinuria to the CV risk prediction was equal, or even greater, than any traditional CV risk factor. Moreover, for eGFR, a cut-off point of 60 mL/min/1.73m^2^ has been identified as the level below which the CV risk starts to increase, while there is no specific threshold for proteinuria. This means that an increase in proteinuria, even within the normal range, confers CV risk.

**Figure 2:**
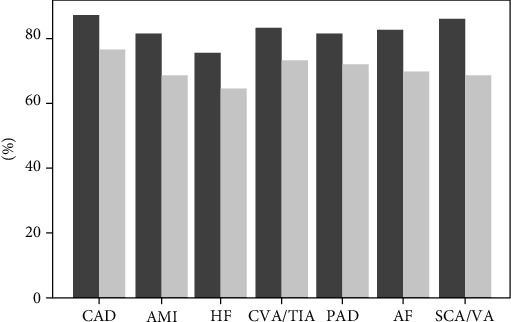
Two-year survival (%) of patients with cardiovascular disease (CVD) by chronic kidney disease (CKD) status. Columns in dark gray depict patients without CKD whereas columns in light gray depict patients with CKD. AF: atrial fibrillation; AMI: acute myocardial infarction; CAD: coronary artery disease; CVA/TIA: cerebrovascular accident/transient ischemic attack; HF: heart failure; PAD: peripheral arterial disease; SCA/VA: sudden cardiac arrest and ventricular arrhythmias. Data source: United States Medicare Population [30].

These data suggest that eGFR and proteinuria should be considered before estimating the CV risk in patients with CKD, especially if considering that the already available risk scores, such as the Framingham or the Atherosclerotic Cardiovascular Disease (ASCVD), failed in predicting CV risk in CKD [6, 7, 21]. The linkage between CV disease and CKD measures has also been recently extended to the peripheral vascular disease (PVD). It has been demonstrated that even slight increases in proteinuria, as well as moderate reductions in eGFR, were found significant predictors of PVD (i.e., peripheral artery disease and leg amputation) beyond traditional CV risk factors [24]. Taken together, CV events are prevalent in CKD patients and are also responsible for most of the unfavorable outcomes. In the Kidney Early Evaluation Program (KEEP), which enrolled subjects at high risk of developing CKD, the overall prevalence of CV disease (CVD) was 22.1% and rose to 30-50% in CKD populations of MASTERPLAN (Netherlands), Chronic Renal Impairment in Birmingham, United Kingdom (CRIB), African Americans Study (AASK), and CKD Multicohort [8, 25–28]. Once CKD is established, up to 50% of patients are reported to die of cardiovascular causes over time [29]. Indeed, in the CKD populations of Kaiser Permanente Northwest, a healthcare service of the United States of America (USA), as well as among CKD diabetic and nondiabetic patients in the USA Medicare system, patients have died or developed CV disease with a higher rate than ESKD and the two-year survival probability in patients with previous CV disease was modified by the presence/absence of CKD (Figure 2) [30, 31]. In the Italian CKD Multicohort, which included CKD patients under stable nephrology care, the incidence rates of ESKD and CV events before ESKD were similar (5.26 vs. 4.52 per 100/pts/year), thus confirming that the CV risk remains a major complication for these patients [5].

Hence, the presence of kidney-specific mechanisms contributes to the raised CV risk beyond traditional risk factors and individual comorbidities. It has been shown that in CKD the expression of endothelial nitric oxide synthase is downregulated [32]. This mechanism has been hypothesized as the main cause of endothelial dysfunction in CKD patients in association with the increased levels of asymmetric dimethylarginine (ADMA). Indeed, ADMA acts by inhibiting generation of nitric oxide and increasing systemic vascular resistance and blood pressure [33]. The endothelial stretch and the increase in ADMA lead to an impairment in coronary vascular resistance and left ventricular hypertrophy [34]. Moreover, although arterial hypertension is present in a large number of CKD patients, it seems that the renal anemia and the increased vascular stiffness mainly contribute to the onset of left ventricular hypertrophy in combination with the endothelial dysfunction [35]. CKD also causes dyslipidemia. In the presence of impaired kidney function, an excessive oxidation of low-density lipoprotein (LDL) cholesterol has been observed together with a defective high-density lipoprotein (HDL) function. The lipid profile becomes, thus, atherogenic [36, 37]. Accordingly, an observational analysis of 1,162 subjects who died between 1988 and 2005 in a suburban community adjacent to Fukuoka City, in southern Japan, showed that the entity of coronary artery stenosis was raised from state I-II to stage V of CKD and that the vascular stenosis was attributable to a worsening atherosclerosis in advanced stages of CKD [38]. CKD is also associated with the presence of systemic inflammation which is, in turn, a trigger for CV damage. The increased oxidative stress and accumulation of toxins, normally excreted in the case of normal kidney function, favor the onset of an imbalance of inflammatory factors. In CKD patients, levels of IL-6 and matrix metalloproteinases (MMPs) have also been found to have increased [39, 40]. Interestingly, it has been shown that MMPs play an important role in expanding inflammatory response and in the inflammation and rupture of atherosclerotic plaques [40]. Another key factor of endothelial dysfunction is proteinuria (or albuminuria). A group of researchers from the Steno Memorial Hospital, in Denmark, described for the first time in 1989 that in diabetic patients with proteinuria the presence of proteinuria was strongly associated with raised levels of von Willebrand factor and transcapillary escape rate of fibrinogen, thus testifying that proteinuria is most likely a marker of systemic vascular damage [41]. Further evidence has confirmed that proteinuria exerts prominent toxic effects on all parts of the nephrons including the renal tubules, thus feeding a vicious circle that moves from kidney to systemic damage [42, 43]. Patients with impaired kidney function present a deficiency in vitamin D, because of the weakened function of the 1*α*-hydroxylase, a renal enzyme which converts the vitamin D precursor to the active hormone. Many studies suggested that vitamin D deficiency is associated with CV risk since the vitamin D pathway directly works in modifying cardiac function [44, 45]. Other factors have been considered as CV risk factors in the early phase of CKD, such as hyperphosphataemia, parathyroid hormone (PTH), and leptin which worsen atherosclerosis, vascular calcifications, and cardiorenal prognosis [45–47].

## 3. Rationale to Incorporate Novel Biomarkers of CV Risk in CKD Patients

Owing to the great burden of CV events in patients with CKD, much effort has been initiated to improve prognosis of these patients. One strategy, which we previously mentioned, is to test novel drugs that would probably represent the best possible treatment in the near future. In this context, SGLT-2i have been shown to reduce the rate of CV events in patients with CKD and diabetes [13, 14] and the results were so promising to the point that new trials have been started testing the effect of SGLT-2i in patients with nondiabetic CKD (ClinicalTrials.gov identifier: NCT03036150). One major concern of these trials is that they answer the question whether one treatment is able to reduce on average the CV risk compared with the standard treatment (control group) without considering the individual response to treatments. Indeed, a variability in response has already been described for drugs intervening in the renin-angiotensin-aldosterone system (RAAS), but also with respect to SGLT-2i, thus meaning that a consistent proportion of patients continue to remain at increased risk if the response to treatment is suboptimal. Another strategy that has been considered is to evaluate, develop, and implement novel biomarkers of CV risk. Biomarkers may improve the management of CKD patients in several ways. Although the increase in proteinuria and the falling of eGFR define CKD, their presence is often the marker of an already established and possibly irreversible kidney damage. In this context, novel biomarkers would be desirable for several reasons:

1. Novel biomarkers that are able to anticipate the diagnosis of kidney damage (at early stage of the disease) would be extremely useful in clinical practice since they help in adopting timely strategies to prevent the progression of kidney disease and CV risk
2. Novel biomarkers can reveal aspects of kidney disease that are not directly captured by eGFR or proteinuria, for example, by informing about the degree of fibrosis, renal inflammation, or tubular damage
3. The combination of the novel biomarker measurement and renal biopsy could be useful in the case whether eGFR and proteinuria are noninformative, such as in nonproteinuric CKD [47]
4. Novel biomarkers must be studied in those fields of research where therapeutic strategies are not yet adequately improved. For example, it has been shown that, among CV diseases, CKD patients are more likely at risk of developing HF than CAD, probably due to left ventricular hypertrophy and the impaired preload that are commonly observed in advanced CKD [21]. It is thus remarkable that, since proteinuria and eGFR may be suboptimal in predicting HF, the role of novel biomarkers in anticipating the clinical diagnosis in order to plan proper therapeutic strategies would be determinant
5. Novel biomarkers could also reveal more information on pathophysiological mechanisms of kidney and CV damage
6. The assessment of clinical utility of biomarkers in large cohorts with proper follow-up is essential in order to understand whether a specific biomarker can be transportable to clinical practice, since it would help to improve monitoring the disease trend over time (prognostic biomarker) or predicting the individual response to a treatment or intervention (predictive biomarker)

## 4. Principally Investigated CV Biomarkers in CKD

Biomarkers have been differently classified in previous available studies. As far as we know, at least three classification systems exist [48–50]. The first considers the anatomic origin or the mechanisms of damage and thus identifies kidney and cardiac markers [49]. A second classification encompasses filtration markers, namely, biomarkers that give a better estimation of GFR as compared to creatinine eGFR and nontraditional biomarkers that were derived from imaging techniques (i.e., coronary artery calcium score) or laboratory measurements [48]. A third classification is based on the clinical “intended use” of the biomarker and distinguishes prognostic and predictive biomarkers [50, 51]. A prognostic biomarker is used to identify the likelihood of the patient to develop a clinical outcome regardless of treatment. Indeed, it can be evaluated in untreated patients or patients who undergo heterogeneous treatments that often happen under the standard of care. Such a measure may improve the physician's ability to identify patients with a poor prognosis. On the other hand, predictive biomarkers are used to determine whether the patient is likely to benefit from a specific treatment. In this context, the clinical benefit is interpretable as either a good response to a drug that can be used if the biomarker is positive or, alternatively, a resistance to the same drug that can save a patient from drug toxicity or pointless side effects. Since we are interested in the clinical utility of the biomarkers, we adopt and follow the latter classification.

### 4.1. Prognostic Biomarkers

In patients with already established CKD, many biomarkers have been shown to improve prediction of CV events. The use of cystatin C to estimate GFR (eGFR_cys_) was able to refine risk stratification of CKD patients as compared to creatinine-based GFR (eGFR_crea_) [1]. eGFR_cys_ affords estimates of kidney function levels that are slightly different from those estimated by eGFR_crea_. A meta-analysis of the CKD Prognosis Consortium showed that the reclassification of patients according to eGFR_cys_ versus eGFR_crea_ is accurate in the sense that patients with lower and higher eGFR_cys_ than eGFR_crea_ levels were, respectively, at higher and lower risk for all endpoints, including CV events [52]. *β*_2_-Microglobulin is another filtration marker that was found to improve prediction of CV events to an extent similar to cystatin C [53]. Strong pieces of evidence toward the utility of cardiac troponins (high-sensitivity cardiac troponin (hs-cTnT)) and natriuretic peptides (N-terminal pro-B-type natriuretic peptide (NT-proBNP)) have been recently published [54, 55]. Blood levels of hs-cTnT and NT-proBNP are routinely used for diagnosing CAD and HF, respectively, and reflect subclinical abnormalities in the heart. Interestingly, in CKD patients, both hs-cTnT and NT-proBNP are more consistently associated with the development of HF than CAD over time. More importantly, this association is true even after accounting for the kidney function level which *per se* alters the serum concentrations of the two biomarkers [56]. The importance of such evidence is enormous when considering that HF is the most represented CV disease in CKD patients and for whom the two kidney measures of CV risk, proteinuria and eGFR, show a suboptimal prediction. The clinical implication is also relevant as these novel biomarkers could be used in the future to identify CKD patients at increased CV risk who could be prescribed with preventive treatments (e.g., statins and/or aspirin therapy) [57]. In the context of HF, two further biomarkers are of particular interest: soluble suppressor of tumorigenicity (sST2) and galectin-3. sST2 is a protein produced by the endothelial cells lining the left ventriculus in response to mechanical strain. It has shown to have an incremental value to NT-proBNP to predict deaths and hospitalizations due to HF, irrespective of kidney function [58]. Galectin-3 is a member of the *β*-galactoside-binding lectin family that interacts with laminin, synexin, and other extracellular matrix proteins. In observational analyses which included patients with HF, serum galectin-3 levels were independent predictors of hospitalizations due to HF and CV mortality, regardless of kidney measures (proteinuria and eGFR) [59, 60]. Markers of inflammation or tissue remodeling have also sparked interest in assessing CV risk in CKD patients. Among these, levels of MMPs have been considered as possible biomarkers. Serum levels of MMP-2, MMP-8, and MMP-9 have been found increased in CKD patients and diabetic patients, being correlated, respectively, with serum phosphate (MMP-2), fibroblast growth factor-23 (FGF-23), and the degree of proteinuria (MMP-8 and MMP-9), two relevant predictors of oxidative stress and CV risk [61–63]. Moreover, MMP-2 has been directly correlated with vascular calcification, atherosclerotic plaque rupture, and carotid intima-media thickness (cIMT), thus playing an important role in atherogenesis [64]. Higher serum levels of MMP-9 and tissue inhibitor of metalloproteinases-1 (TIMP-1) are involved in the pathogenesis of left ventricular hypertrophy by cleaving intracellular myosin filaments [65, 66]. Several MMPs, such as MMP-2, MMP-3, and MMP-9, are also implicated in the pathogenesis of vascular aneurysm and their levels after surgical interventions for lower extremity bypass were an independent predictor of CV death [40]. All these mechanisms of damage are made even worse by the presence of an inflammatory milieu in patients with CKD and by the raised serum concentration of MMPs due to the reduction of GFR. The assessment of measures of CV disease process has been also evaluated as biomarkers of CV risk in CKD. Among these, the coronary artery calcium (CAC) score has been used. CAC score is computed using either an electron beam or multidetector cardiac computed tomography (CT). Afterward, a semiautomated tool called Agatston score is used to create a risk score based on the degree of plaque densities and their areas in all coronary arteries [67]. CAC score has shown to be a reliable predictor of atherosclerotic CV disease among the general population and in patients with moderate and advanced CKD beyond traditional risk factors and with a discrimination ability that is greater than of other filtration markers such as cystatin C [48, 68].

### 4.2. Predictive Biomarkers

One fascinating and advantageous aim of the biomarkers is to identify individuals who will likely respond to a drug of interest. These biomarkers are commonly defined “predictive” biomarkers. The baseline level of a predictive biomarker could also change over time (dynamic predictive biomarker) as a treatment-induced effect, so it can be used for monitoring the course of the disease and its treatment efficacy [50]. Predictive biomarkers can be genes, proteins, metabolites, or others. The most used predictive biomarker in nephrology is the presence of proteinuria. Several clinical trials have shown in the past three decades that the drug-induced reduction in proteinuria is associated with a protection from CV risk over time both in diabetic and nondiabetic CKD patients [12–14, 69, 70]. Treatments tested in these trials were disparate and included antihypertensive, diuretics, and oral hypoglycemic agents. However, the common pieces of evidence derived from these studies were that (1) the magnitude of treatment effect, i.e., risk reduction for fatal and nonfatal CV events, was greater in patients with proteinuric CKD phenotype as compared to those without CKD and (2) the extent of CV risk reduction after interventions was strictly correlated with the reduction in proteinuria [69, 70]. Two post hoc analyses of clinical trials enrolling CKD patients, the Reduction in Endpoints in Noninsulin-dependent diabetes mellitus with the Angiotensin II Antagonist Losartan (RENAAL) and the Irbesartan Diabetic Nephropathy Trial (IDNT) study, have shown that the greatest protective effect was found in patients with the larger reduction of proteinuria after 6 months from randomization visit that corresponds to the start-of-treatment visit [70, 71]. There is now a general agreement, confirmed by KDIGO guidelines, that proteinuria should be measured in CKD patients to monitor the progression of the disease. However, although further studies are needed to establish how often it should be measured and what the correct threshold that confers a strong protection against CV disease, it is reasonably accepted based on previous trials that a 30% reduction of proteinuria after 6 months is a sufficient target [42, 43]. Presently, in clinical research in nephrology, additional predictive biomarkers that are able to predict the response to nephroprotective treatments beyond proteinuria would allow to better control the CV risk and refine the treatment decision toward “the right drugs for the right patient” perspective. There is interesting evidence that MMPs could play a predictive, other than prognostic, role in CKD patients [40]. In fact, a reduction in serum concentration of MMPs in response to the antibiotic doxycycline and the nonselective inhibitors of MMPs Batimastat and Marimastat has been associated with a reduction of detrimental vascular tissue remodeling and to a significant reduction of proteinuria in patients with CKD [40, 72]. Even more importantly, the novel SGLT-2i medications, which have been widely demonstrated to reduce the cardiovascular risk in CKD patients in several clinical trials, may exert part of their CV and renal risk reduction effect through a mechanism that is independent from the level of proteinuria and is possibly based on the activation of an endogenous inhibitor of MMPs, the reversion-inducing cysteine-rich protein with kazal motifs (RECK) [73]. This is important for improving clinical trial design in CKD, since novel drugs may be also tested in nonproteinuric subjects, which represent a nonnegligible part of the CKD cohort [47]. A growing body of evidence is emerging around the role of renal resistive index (RI) as a dynamic biomarker of CV risk. It is well known that impaired RI levels reflect both kidney and systemic vascular damage [74, 75]. Moreover, RI also predicts CV events in high-risk patients regardless of eGFR and proteinuria [76]. Interestingly, recent studies showed that RI can change over time and in response to treatments. Solini and colleagues have demonstrated that the SGLT-2i dapagliflozin improves endothelial function, vascular damage, and RI in type 2 diabetic patients [77]. A similar effect is determined by the RAAS inhibitors [78]. Hence, novel studies should assess whether the dynamic changes in RI and its trajectory over time could influence prognosis. An insertion/deletion polymorphism of the angiotensin-converting enzyme gene was able to predict the response to losartan in type 2 diabetic patients enrolled in the RENAAL study trial [79]. This evidence was also replicated in nondiabetic patients, thus testifying that intrarenal RAAS activity has a role in CV risk prediction as well as in response to treatment prediction [80]. Among complex biomarkers, a panel of 185 serum metabolites, including amino acids, energy/sugar lysophosphatidylcholines, phosphatidylcholines, and sphingomyelins, was analyzed to select a subset of metabolites, which predicts accurately the response to the angiotensin receptor blocker (ARB) therapy in diabetic patients. That prediction ability was also independent from main confounding covariates such as age, gender, eGFR, and proteinuria [81]. Similarly, another classifier has been developed from the PREVEND study, using plasma proteomic profiles which have been shown to predict the change in albuminuria stage and to improve the prediction ability of standard risk factors like albuminuria, eGFR, and RAAS inhibitor use [82]. A summary of the principal prognostic and predictive biomarkers of cardiovascular risk in chronic kidney disease patients is provided in Table 1.

## 5. Strategies for Implementing Novel Biomarkers of CV Risk in the CKD Setting

CV disease is a major complication of CKD patients. Despite the introduction of novel treatments and a stricter monitoring of patients, the frequency of CV fatal and nonfatal events remains disproportionately high [84, 85]. Moreover, the risk of CV events among these patients equals or even overcomes the competing risk of CKD progression or ESKD [5]. The correct detection, assessment, and implementation of novel biomarkers may certainly support the aim of improving CV risk management in the CKD setting. As we previously discussed, several biomarkers have been demonstrated to play a prognostic or predictive role but just a few biomarkers have made it from the discovery phase to clinical use. With the exception of cystatin C, whose adoption allowed a refinement in the estimation of GFR and CV risk prediction, the risk markers widely used currently in CKD patients are eGFR and proteinuria. Although they convey a great part of information for individual prognosis and treatment decision as well, several concerns have been recently raised. Yoshio Hall and Jonathan Himmelfarb, in a recent Editorial, reported in the *Clinical Journal of the American Society of Nephrology*, defined the eGFR/proteinuria-based classification a “reductionist” approach, since it does not consider that CKD could manifest through a myriad of clinical and histological phenotypes and that each renal diagnosis deserves a proper comprehensive investigation [86]. The major limitations to the development of previous biomarkers are represented by the small sample sizes, the heterogeneous results from a specific biomarker assessment, and the lack of result validation [87]. The framework for the development of a prognostic biomarker includes a series of steps [88]. Briefly, to determine if a biomarker improves the clinical prediction on top of already available variables included in risk prediction models, it is recommended to report model *calibration*, meaning that the event rates predicted by the model correspond to those rates observed in a clinical setting; the *significant association* of the biomarker with a clinical outcome that should be independent from other main confounders (the effect size of the biomarker with the outcome after multiple adjustments and the *p* value should be considered); *discrimination*, a measure according which a model has a good performance if it classifies at high-risk patients who develop the outcome of interest and at low risk those who do not. Although sensitivity and specificity are the proper measures for a precise threshold of the biomarker, a summary measure that depicts sensitivity and specificity for all possible thresholds is the Receiver Operating Characteristic (ROC) curve. It is thus suggested to present the ROC derived from the model together with the Area Under the Curve (AUC) that in these cases is also called *c*-statistic [89]. If the model with the biomarker *c*-statistic is significantly higher than the model without the biomarker, it could be clinically useful; *reclassification measures*, such as net reclassification improvement (NRI) and integrated discrimination index (IDI). Indeed, if the prediction model with the standard covariates (e.g., a model with eGFR and proteinuria in nephrology) accounts for most of prognostic information, it is hard to find a significant improvement of *c*-statistic, following the statement “it is hard to improve an already good thing.” For this reason, measures of reclassification could give useful information on the frequency (%) of patients that are reclassified in the true risk category (lower or higher) with the addition of the new biomarker as compared to the traditional model [90]. During all these phases, it is important to keep in mind the intended use of the biomarker (e.g., what kind of outcome it may predict) and the clinical setting (CKD, general population, and high-risk population), since different clinical settings may give disparate results and the variables that influence the effect size of the biomarker. To this aim, it is useful to run subgroup analyses (e.g., by age, gender, race, eGFR, or proteinuria categories). After computing and depicting the measures of performance, a crucial step forward is to validate biomarker performance. Indeed, if biomarker performance is measured on the same cohort from which it was developed, this performance is likely overestimated. Two strategies to assess a correct validation and avoid overfitting are the internal and external validation [89]. The internal validation consists in splitting the cohort in multiple samples so that it is possible to develop and validate the biomarker in different samples of the same cohort. Alternatively, cross-validation and bootstrap-based methods can be used [91]. External validation allows one to transport and apply the model to different populations. The biomarker performance may be poor in other populations because the baseline characteristics (frequency of diabetes, CV disease, and degree of kidney impairment) are often different, thus varying the baseline risk of the new population. However, strategies to recalibrate and adapt the performance measures to the new population are applicable [92]. Hence, external validation is considered the most effective way to validate a biomarker. Predictive biomarker performance should be assessed following the same scheme used for the prognostic biomarkers. However, predictive biomarkers are also useful in research to select patients for new trials testing drugs for CV protection. A strategy that follows this concept is the adaptive enrichment design [83]. This design consists in enrolling patients who respond to a drug rather than randomize all the population irrespective of a response/no response. Advantages from this strategy are several. Firstly, patients under study would avoid a long period of ineffective therapy if they were nonresponders. Secondly, since all the patients are treated with the study drug before randomization (the run-in period), the treatment effect is estimated in a proper fashion. Finally, such a design is close to clinical practice since clinicians are used to continuing a treatment only if patients respond to that treatment. Predictive biomarkers could be also used to better understand the phenomenon of variability in response to treatment. The crossover studies and even the single-patient trials, the so-called *n-of-1*, may help to answer this important question. Indeed, in these study designs, patients are randomized to 2 or more sequences of different drugs interspersed with a washout period. With such a design, by measuring a panel of biomarkers before starting each treatment, it is possible to assess what are the characteristics of a patient who responds to the first treatment and does not respond to the second treatment or vice versa. This could also lead in the future to dose a biomarker before selecting the correct treatment as well. One example of such a crossover study is the ROTATE trial (ClinicalTrials.gov identifier: NCT03504566); the results of which are eagerly expected in 2021.

## 6. Conclusions

Owing to the global dimension of CKD and the high prevalence of CV disease in this setting, great effort is currently ongoing with the aim of reducing CV residual risk. One important strategy that can be pursued to this aim is to develop reliable prognostic and predictive biomarkers. In fact, eGFR and proteinuria, despite their great importance, have shown suboptimal performance in predicting several CV outcomes in CKD patients such CAD and heart failure [93]. Predicting the response to treatments is another important scope of clinical research since it allows to individualize therapies, to improve the clinical trial design, and to better comprehend the variability in the response to different treatments. The implementation of novel biomarkers of CV risk from the discovery to clinical practice should follow a rigorous methodology so that it would be possible to improve the management of patients by clinicians.

## Figures and Tables

**Table 1:** Summary of the principal prognostic and predictive biomarkers of cardiovascular risk in chronic kidney disease patients.

Biomarkers	Characteristics	Prognostic value	Predictive value
Cystatin C	Protein produced by all nucleated cells mainly used as marker of kidney function	Cystatin C improves the estimation of eGFR and risk prediction of CV events; it also allows to reclassify patients into more accurate CV risk categories [52]	—
*β*2-Microglobulin	Component of MHC class I molecules and expressed on all nucleated cells in humans	Improves risk prediction in CKD patients beyond traditional risk factors [53]	—
hs-cTnT	Regulatory protein that is integral to cardiac and skeletal muscle contraction	Improves the risk prediction of CV events, particularly heart failure regardless of the level of kidney function [54–56]	—
NT-proBNP	Prohormone with a 76-amino acid N-terminal inactive protein	Improves the risk prediction of CV events, particularly heart failure regardless of the level of kidney function [54–56]	It has been used as predictive biomarker in the SONAR trial during the run-in phase, in order to exclude patients with sodium retention after treatment with atrasentan [83].
sST2	Member of the IL-1 receptor family, which is produced by cardiomyocytes and cardiac fibroblasts	It is delivered in response to mechanical stress conditions and showed incremental prediction ability (over NT-proBNP) for HF-related death and hospitalizations [58]	—
Galectin-3	30 kDa protein that contains a carbohydrate-recognition-binding domain that enables the linkage of *β*-galactosides	In patients with already established CV disease, galectin-3 is an independent predictor of hospitalizations and death due to CV causes [59, 60]	—
MMPs	Six families of zinc-containing endopeptidases that are involved in regulating tissue development and homeostasis	Serum MMP-2, MMP-8, MMP-9, and TIMP-1 are associated with atherogenesis, the severity of kidney damage, and the onset of left ventricular hypertrophy and peripheral vascular disease [61–66]	MMP levels are modified by selective and nonselective drugs. Changes in MMP levels have been associated with a reduction of CV risk [72, 73].
CAC	CAC is a score measured at cardiac TC based on the entity of calcium depositions on artery plaques.	Improves risk prediction in CKD patients beyond traditional risk factors [48, 68]	—
eGFR_crea_	eGFR_crea_ is an estimation of the kidney function level based on serum creatinine, age, gender, and race.	A reduction of eGFR is a potent predictor of CV endpoints, regardless of age, gender, and other risk factors [1, 2, 5, 8, 22, 23]	Although a treatment-induced reduction of eGFR is considered a surrogate endpoint of ESKD, the predictive role of eGFR change for CV risk is still controversial [67].
Proteinuria	Presence of an abnormal quantity of proteins in urine; it is considered the principal marker of kidney damage.	The increase in proteinuria is strongly associated with the onset of fatal and nonfatal CV events [1, 2, 5, 8, 21, 22]	In clinical trials, patients who develop a significant reduction in proteinuria during the first months after treatment were protected against CV events over time [12–15, 69–71].
RI	Renal resistive index is a sonographic index of intrarenal arteries defined as (peak systolic velocity − end diastolic velocity)/peak systolic velocity.	Raised RI levels above have been shown to predict CV events in hypertensive and CKD patients [75, 76]	Medications as RAAS inhibitors and SGLT-2i reduce RI levels over time and improve vascular damage [77, 78].
ACE ID/DD	Insertion (I)/deletion (D) polymorphism of the angiotensin-converting enzyme (ACE) gene influences the circulating and renal activity of RAAS.	The D allele patients showed a poor CV prognosis in the RENAAL trial [79]	Patients with DD genotype, despite being at high risk of CV events, showed the better response to losartan in the RENAAL study [79].
Classifiers	A classifier is the combination of the informative markers which is able to classify patients according to their risk of developing an outcome or likelihood of response to a treatment.	—	A panel of 185 metabolites and a proteomic-based classifier have shown to predict the proteinuric response to RAAS inhibitors [81, 82].

## Data Availability

The underlying data supporting the results of our study can be asked from the corresponding author.
